# Partnering and parenting transitions in Australian men and women: associations with changes in weight, domain-specific physical activity and sedentary behaviours

**DOI:** 10.1186/s12966-020-00989-6

**Published:** 2020-07-08

**Authors:** Jing Tian, Kylie J. Smith, Verity Cleland, Seana Gall, Terence Dwyer, Alison J. Venn

**Affiliations:** 1 Menzies Institute for Medical Research, University of Tasmania, 17 Liverpool Street, Hobart, Tasmania 7000 Australia; 2 The George Institute for Global Health, University of Oxford, Wellington Square, Oxford, UK

**Keywords:** Weight, Physical activity, Sedentary behaviour, Marriage, Parent, Young adult

## Abstract

**Background:**

Partnering and parenting are important life-stage transitions often accompanied by changes in social networks, roles and responsibilities. There have been no longitudinal studies examining associations of partnering and parenting with changes in domain-specific physical activity (PA) and sedentary behaviours, and our understanding of whether these transitions are associated with weight change is limited.

**Methods:**

Two thousand one hundred and twenty-four Australian adults from a national cohort (mean age 31.7 (2.7) years, 47.5% male) completed questionnaires at baseline (2004–06) and follow-up (2009–11), reporting marital and parental status. Weight (kg) was measured at baseline and self-reported at follow-up. PA and sedentary behaviours (sitting and television (TV) viewing) were self-reported in a subset (*n* = 1221). Linear regression estimated the longitudinal associations of parenting and partnering transitions with PA, sedentary behaviours and weight at follow-up, adjusted for baseline value of the respective outcome variable, age, education, follow-up duration and other life-stage transition.

**Results:**

During the 5-year follow-up, 17.3% men and 12.9% women partnered, and 27.3% men and 19.1% women had their first child. Compared to staying not partnered, partnering was associated with an increase in total PA (177.5mins/week, 95% Confidence Interval (CI) 18.0 to 337.0) among men and a greater weight gain (2.2 kg, 95% CI 0.6 to 3.7) among women. Compared to remaining child-free, having a first child was associated with greater reductions in total PA (− 123.9mins/week, 95% CI − 248.8 to 1.1) and TV viewing time (− 27.0mins/day, 95% CI − 50.6 to − 3.3) among men. Women who had their first child had greater weight gain (1.4 kg, 95% CI 0.1 to 2.7) but spent less time sitting (− 103.8mins/day, 95% CI − 135.5 to − 72.1) than those remaining child-free. For women, having additional children was associated with less sitting time (− 39.4mins/week, 95% CI − 66.0 to − 12.8) than having the same number of children.

**Conclusions:**

Partnering was associated with an increase in men’s total PA and women’s weight. Transitions into parenthood with a first child or additional children were associated with potentially health-impairing changes in weight and PA, but health-promoting changes in sedentary behaviours. Future PA promotion strategies should pay attention to men who had their first child to mitigate declining total PA.

## Background

Becoming partnered and having children are two important life-stage transitions that typically occur in young adulthood. They are accompanied by major changes in social networks and roles, responsibilities and expectations, which may be associated with changes in lifestyle behaviours, such as physical activity (PA) and sedentary behaviour. Over recent decades, transitions to partnership and parenthood have been occurring at later ages. Data from the Australian census in 2016 showed that the median age at first marriage was 30.3 years for males and 28.7 years for females [1], rising by 4.0 and 4.5 years since 1989 respectively [2]. The average age of first-time mothers also increased, from 26.2 years in 1993 [3] to 29.2 in 2017 [4].

Previous studies, with varying follow-up durations, generally show that partnering is associated with weight gain, while divorce/separation/widowhood is associated with weight loss [5, 6]. However, the findings differed by sex. A 10-year United States (US) study of 9043 men and women found that unmarried women who became married gained more weight than women who remained married at both baseline and follow-up, and men who stayed divorced/widowed and men who became widowed lost more weight than men who remained married at both times [6]. In contrast, one 20-year follow-up of 3347 US men and women reported the association of partnering transitions with weight change was not contingent on sex [5].

There is a large amount of evidence that transition into parenthood is associated with weight gain among women and the magnitude of weight gain is positively related to parity [7]. For example, a cross-sectional US study of 4523 middle-aged couples reported a 7% increase in obesity risk for each additional child among women [8]. Although the social and biological features of parenthood differ for women and men [9], very few longitudinal studies have quantified weight changes following parenting transitions among men. Using 15-year longitudinal data, one study found that becoming a father was associated with accelerated weight gain [10]. A similar association was reported in a 20-year longitudinal study of young men [11]. However, these two studies have limitations: one included a wide age range of participants (24–96 years) [10] and both failed to control for important confounders [10, 11], such as the aforementioned partnering transitions.

Associations between partnering transitions and change in PA have been reported by several prospective studies with conflicting findings. Some reported that people who married or started to live as married tended to decrease PA relative to those remaining not partnered [12–16], while others found increased [17] or no significant difference [18–21] in PA. Direct comparison of the results in these studies is difficult because they used different PA measurements varying from one question on leisure and work PA [17], two or three questions on moderate and vigorous leisure time PA in the past week [12–14], leisure time PA in the past year [16, 18] and total moderate and vigorous PA from three questions [21]. In addition, the socio-demographics of participants varied widely across studies. Some of them examined participants aged in their twenties [12–14, 18], while others examined a far wider age range of 18–83 [22] and 25–75 years [17] and mid-aged to elderly [16, 20]. Further, some of them included women only [12–14, 20], one included men only [16] while others included both [17–19, 22]. These characteristics are important as the impacts of partnering transitions on PA were suggested to differ by age and sex [15]. The association between becoming partnered and changing PA became weaker with increasing age and shifted from negative to positive in men [15]. The effect size decreased with increasing age in women as well but stayed negative with older age [15].

Having children has been shown to have negative associations with PA in young women [12–14]. For example, a 3-year follow-up of Australian women aged 22–27 years found that women having their first child during follow-up were 45% more likely to decrease PA [12]. Only one study has explored the associations between parenting transitions and changing PA in men [18]. This study by Hull et al. [18] followed 638 young adults aged in their mid-twenties (48% male) for 2 years and found different patterns between parenting transitions and changing leisure time PA for men and women. Having a first child was significantly associated with decreased leisure time PA in both men and women compared with remaining child-free, while having additional children was associated with decreased leisure time PA among women but not men compared with having the same number of children [18]. This study also had limitations including small sample, poor generalisability and inadequate control of confounders (e.g. did not adjust for parenting transitions in partnering transition analyses).

Total PA is constructed from different domains relating to occupation, transport, domestic and leisure time PA. The intensity, duration and frequency of each domain are used to estimate total PA. Previous prospective studies have often focused on a limited number of domains, mainly leisure time PA [12–14, 16, 18]. It remains unclear how partnering and parenting transitions associate with other domains and total PA. Data of this kind are important as they will help establish whether interventions that target specific PA domains may be required. Further, no study has explored the associations of partnering and parenting transition with changing sedentary behaviours.

Using data from a large population-based national cohort in Australia, this study aimed to examine 1) whether partnering and parenting transitions were associated with changes in weight, domain-specific PA, total PA and sedentary behaviours over a 5-year period, and 2) whether the associations differed by sex. We hypothesised that 1) participants who became partnered or had children during the 5-year follow-up would gain weight and have healthier PA and sedentary behaviours than those who stayed not partnered or child-free and 2) these associations differ between men and women in effect size.

## Methods

### Study design

A prospective cohort study.

### Participants

Participants were from the Childhood Determinants of Adult Health (CDAH) study [23], a follow-up of 8498 participants from the 1985 Australian Schools Health and Fitness Survey (ASHFS), which comprised a nationally representative sample of Australian school children aged 7–15 years. During 2002–4, 6840 participants were traced and 5170 agreed to participate in the CDAH study. The first follow-up was conducted in 2004–6 (CDAH-1, baseline in this study) where 3975 participants aged 26–36 years completed questionnaires and 2410 attended one of 34 study clinics held around Australia for physical measurements. In 2009–11, the second follow-up (CDAH-2, follow-up in this study) collected data from 2820 participants aged 31–41 years via telephone, mail or online survey.

### Marital status and partnering transitions

At baseline and follow-up, participants were asked to report their current marital status (single, married/living as married, and separated/divorced/widowed). This information was used to create a four-category variable of partnering transitions: “stayed not partnered” (single or separated/divorced/widowed at both baseline and follow-up), “became partnered” (single or separated/divorced/widowed at baseline, and married/living as married at follow-up), “stayed partnered” (married/living as married at both baseline and follow-up), and “became separated/divorced/widowed” (married/living as married at baseline, and separated/divorced/widowed at follow-up). Those who became partnered were compared to those who stayed not partnered. Those who became separated/divorced/widowed were compared to those who stayed partnered.

### Parental status and parenting transitions

At follow-up, participants reported how many biological children they had and the month and year of birth for each child. The date the participant completed the baseline questionnaire was used to determine whether each child had been born before or after the baseline assessments. Participants were then classified into one of four groups: “stayed child-free, “had first child”, “had additional children”, and “same number of children”. If participants had their first child plus additional children since baseline, they were classified into the group “had first child”. Those who stayed child-free were compared to those who had their first child. Those who had additional children were compared to those who had the same number of children.

### Anthropometric measurements

At baseline, weight (kg) and height (cm) were objectively measured at study clinics for most participants (*n* = 2410) by trained clinic staff. A subsample (*n* = 1185) of these participants also self-reported their weight and height before measurements were taken by clinic staff to assess the accuracy of self-reported values. Clinic weight and height measures were used to predict the difference between self-reported and clinic weight and height. A correction factor that gave estimates of clinic weight and height from self-reported values was obtained from a linear regression model [24]. Participants who did not visit a study clinic (*n* = 1565) self-reported their weight and height and the correction factor was applied to adjust for error. Body mass index (BMI, kg/m^2^) was calculated from height and weight. The agreement between self-reported and clinic BMI categories was high in men (κ = 0.80) and women (κ = 0.82) [24].

Weight was self-reported at follow-up. Adjusted weight values were calculated using the correction factor applied at baseline [24]. BMI was calculated using adjusted height at baseline and adjusted weight at follow-up.

### PA and sedentary behaviours assessment

Domain-specific PA and time spent sitting were measured using the long version of the International Physical Activity Questionnaire (IPAQ-L) [25]. Participants were asked to report the total time (mins) and frequency (times/week) of occupational, domestic, transport and leisure time PA during the past week. Minutes/week spent in each domain were calculated by multiplying frequency by duration. Times spent doing PA in each domain were summed to estimate the total PA (mins/week). Time spent sitting was reported for a typical weekday and weekend day. The average daily sitting time (mins/day) was calculated by summing time spent sitting on weekdays and weekend days and dividing by seven. Daily TV viewing time (mins/day) in the past week was estimated from self-reported total time spent watching TV, digital video disks, or videocassettes (prevalent at the time the survey was conducted) on weekdays and weekend days as described in detail elsewhere [26].

### Covariates

Socio-demographic information was self-reported at baseline, including age, sex, highest level of education, occupation and diet quality. Diet quality was assessed using a validated dietary guidelines index (DGI) which measures compliance with the 2013 Australian Dietary Guidelines. The DGI has a potential score range of 0–100, with higher scores reflecting better diet quality [27]. Follow-up duration was calculated from the dates the participant completed the baseline and follow-up questionnaires. Parenting transitions were considered as a covariate in the analyses of partnering transitions and vice versa.

### Statistical analyses

Descriptive statistics, including Mean (Standard Deviation (SD)) for continuous variables and % (n) for categorical variables, were used to report the characteristics of participants and changes in marital and parental status, weight, PA and sedentary behaviours. Paired t-tests were used to examine whether mean scores of weight, PA and sedentary behaviours differed significantly between baseline and follow-up. Mean differences in weight, PA and sedentary behaviours at follow-up by partnering and parenting transitions were calculated separately using linear regression, adjusted for the baseline value of the outcome variable. Student t-tests and chi-square tests were used to compare differences in means and proportions of characteristics at the initial 1985 assessment and baseline between respondents and non-respondents.

Covariates were considered as potential confounders if they were causally related to the outcome according to prior knowledge, imbalanced between the exposure groups and caused a change of 10% or more in the estimated effect size when included in a given regression model.

We separated men and women for the analyses because sex differences were expected and of interest. In addition, interactions between sex and partnering or parenting transitions on changes in weight, domain-specific PA and sedentary behaviours were investigated in regression models. A statistically significant sex interaction was present for the association of partnering transition with changes in moderate intensity PA (*P* = 0.014) and sitting time (*P* = 0.002) and was present for the associations of parenting transition with BMI (*P* = 0.030), moderate intensity (*P* = 0.002), occupational (*P* = 0.001) and domestic PA (*P* < 0.001) and sitting time (*P* < 0.001). To be consistent across all models, the analyses were sex stratified.

Sensitivity analyses were conducted using combined multiple imputation (MI) and inverse probability weighting (IPW) to examine the effects of loss to follow-up on the results [28]. Age, sex and school type in 1985 ASHFS were used to impute missing data and the following factors in the 1985 ASHFS were used in the calculation of the weights used in IPW to account for the loss to follow-up: height (cm), weight (kg), arm girth (cm), waist girth (cm), hip girth (cm), sit and reach (cm), sit-ups (number), standing long jump (cm), time spent to complete a 1.6 km run (minutes: seconds), time spent to complete a 50 m run (seconds), area level disadvantage, school enjoyment, school assessed and self-reported scholastic ability [23, 29]. Second, we repeated the weight analyses by excluding women who had their baby in the last 3 months (*n* = 30) as they probably still had some extra weight gain from pregnancy.

All analyses were performed with STATA software, version 12.1 (Stata Corp, College Station, Texas 77,845 USA). A two-tailed *P* value less than 0.05 was considered statistically significant.

## Results

**Fig. 1:**
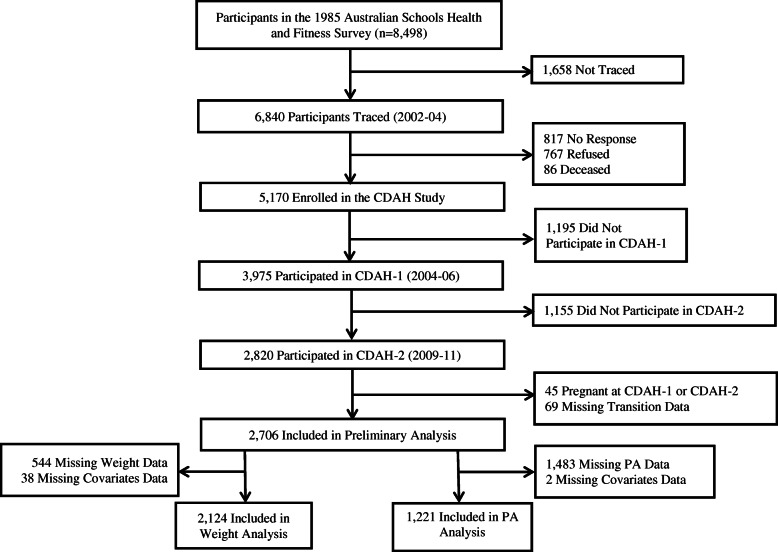
Flow chart of recruitment and retention of participants in the Childhood Determinants of Adult Health Study, Australia, 1985–2011

Analyses were restricted to participants with complete information on the outcomes, exposures and confounders, leading to different sample sizes for the analyses of weight change (*n* = 2124), and PA and sedentary behaviours (*n* = 1221) (Fig. 1).

Compared with participants who were not included in the analyses, those who were included were more likely to be younger, female, more highly educated and employed as professionals or managers, married or living as married and had children at baseline (CDAH-1). There was no statistically significant difference between the two groups in baseline weight.

Using data from the initial childhood assessment, we also explored the extent to which the adult cohort represented the childhood sample. As shown in Table S1, those who were assessed in CDAH-1 (*n* = 3975) tended to be older, more likely to be female, of higher area-level socioeconomic position and healthier than those not assessed (*n* = 4523) as indicated by their greater prevalence of healthy weight status, greater height, and better self-reported health and performance in a range of fitness measures.

The socio-demographic characteristics of participants are shown in Table 1. At baseline, 67.9% of men and 71.8% of women were married or living as married and 42.2% of men and 53.1% of women had children. Compared with the Australian general population of adults aged 25–34 years, a higher percentage of CDAH participants at baseline were married or living as married (55.6% vs. 67.9% for men and 64.0% vs. 71.8% for women) [30] and employed as professionals or managers (33.9% vs. 60.2% for men and 42.7% vs. 51.3% for women) [31]. No significant difference was observed for the proportion classified as overweight or obese (BMI ≥ 25 kg/m^2^) (64.6% vs. 60.9% for men and 43.2% vs. 38.4% for women) [32] and the proportion categorised as child-free (for women only, 49.7% vs. 46.9%) [33].

**Table 1:** Characteristics of participants at baseline (2004–06), Childhood Determinants of Adult Health Study, Australia

Characteristics	Men (***N*** = 1008)^**a**^	Women (***N*** = 1116)^**a**^
Age (years), Mean (SD)	31.8 (2.7)	31.6 (2.7)
Height (cm), Mean (SD)	179.8 (6.79)	166.0 (6.6)
Weight (kg), Mean (SD)	85.3 (14.4)	68.9 (14.7)
Weight status, % (n)		
Normal	39.1 (394)	61.6 (687)
Overweight	46.4 (468)	24.4 (272)
Obese	14.5 (146)	14.1 (157)
Education, % (n)		
Any university education	37.3 (376)	46.6 (520)
Vocational training	36.0 (363)	25.5 (285)
High school only	26.7 (269)	27.9 (311)
Occupation, % (n)		
Professional or manager	60.2 (465)	51.3 (456)
Non-manual	7.2 (56)	27.0 (240)
Manual	29.2 (226)	4.4 (39)
Not in the workforce	3.4 (26)	17.3 (154)
Marital status, % (n)		
Single	29.5 (297)	24.7 (276)
Married/living as married	67.9 (684)	71.8 (801)
Separated/divorced/Widowed	2.7 (27)	3.5 (39)
Parental status, % (n)		
Child-free	57.8 (583)	46.9 (523)
Has child (ren)	42.2 (425)	53.1 (593)

SD, standard deviation; BMI, body mass index

^a^ Because of missing data, numbers do not always equal the total

The changes in marital and parental status, weight, domain-specific PA and sedentary behaviours from baseline to follow-up are presented in Table 2. Over the 5-year follow-up period, 17.3% of men and 12.9% of women became partnered and 27.3% of men and 19.1% of women had their first child. During follow-up, there were statistically significant increases in weight, BMI and domestic PA for both men and women. Occupational PA significantly decreased among men over the 5-year follow-up. For women only, there was a significant increase in moderate intensity PA and a decrease in sitting time over the follow-up.

**Table 2:** Changes in marital and parental status, weight, domain-specific physical activity and sedentary behaviours from baseline (2004–06) to follow-up (2009–11), Childhood Determinants of Adult Health Study, Australia, 1985–2011

Variables	Men	Women
Change in marital status, % (n)		
Stayed not partnered	14.9 (150)	15.3 (171)
Became partnered	17.3 (174)	12.9 (144)
Stayed partnered	64.0 (645)	66.6 (743)
Became separated/divorced/widowed	3.9 (39)	5.2 (58)
Change in parental status, % (n)		
Stayed child-free	30.6 (308)	27.7 (309)
Had first child	27.3 (275)	19.1 (213)
Had additional children	19.4 (196)	19.0 (212)
Same number of children	22.7 (229)	34.2 (382)
Weight (kg), Mean (SD)		
Baseline	85.3 (14.4)	68.9 (14.7)
Follow-up	87.8 (15.2)***^a^	71.4 (15.7)***^a^
BMI (kg/m^2^), Mean (SD)		
Baseline	26.4 (4.1)	25.0 (5.1)
Follow-up	27.2 (4.7)***^a^	25.8 (5.6)***^a^
Total PA (mins/wk), Mean (SD)		
Baseline	738.0 (529.9)	703.9 (462.8)
Follow-up	699.9 (503.1)	741.3 (476.0)
Walking PA (mins/wk), Mean (SD)		
Baseline	262.9 (280.8)	245.4 (243.8)
Follow-up	237.9 (260.9)	243.1 (259.5)
Moderate intensity PA (mins/wk), Mean (SD)		
Baseline	303.4 (240.5)	376.9 (315.7)
Follow-up	306.6 (244.6)	415.8 (328.0)**^a^
Vigorous intensity PA (mins/wk), Mean (SD)		
Baseline	171.8 (222.2)	81.6 (155.5)
Follow-up	155.4 (202.2)	82.4 (129.4)
Occupational PA (mins/wk), Mean (SD)		
Baseline	285.3 (394.8)	146.3 (290.4)
Follow-up	241.6 (355.6)*^a^	127.0 (252.4)
Transport PA (mins/wk), Mean (SD)		
Baseline	109.4 (163.7)	105.4 (150.5)
Follow-up	103.0 (145.9)	103.4 (166.3)
Domestic PA (mins/wk), Mean (SD)		
Baseline	160.6 (167.8)	302.2 (307.4)
Follow-up	188.2 (193.5)**^a^	351.1 (321.4)***^a^
Leisure time PA (mins/wk), Mean (SD)		
Baseline	182.7 (232.2)	150.0 (177.6)
Follow-up	167.1 (195.5)	159.9 (175.3)
Sitting time (mins/day), Mean (SD)		
Baseline	369.9 (180.0)	326.0 (162.5)
Follow-up	364.5 (184.9)	297.3 (159.2)***^a^
TV viewing time (mins/day), Mean (SD)		
Baseline	125.8 (90.6)	102.2 (72.6)
Follow-up	128.2 (92.6)	100.8 (70.0)

SD, standard deviation; BMI, body mass index; PA, physical activity

^a^ There was statistically significant difference compared to baseline value, *P*-value was calculated using Paired t-test

*P ≤ 0.05, **P ≤ 0.01, ***P ≤ 0.001

Weight increased among all partnering transition groups during the 5-year period (Table S2). As shown in Table 3, after adjusting for age, sex, education and weight at baseline, follow-up length and parenting transitions, women who became partnered were, on average, 1.8 kg or 0.7 kg/m^2^ heavier than those who stayed not partnered. This association was absent among men. There was no significant difference in weight and BMI at follow-up between those who stayed partnered and those who became separated/divorced/widowed.

**Table 3:** β (95% CI) for weight, domain-specific physical activity and sedentary behaviours by change in marital status, Childhood Determinants of Adult Health Study, Australia, 1985–2011

Variables	Men			Women		
	n	Model 1	Model 2	n	Model 1	Model 2
		β (95% CI)	β (95% CI)		β (95% CI)	β (95% CI)
Weight (kg)						
Stayed not partnered	150	REF	REF	171	REF	REF
Became partnered	174	0.7 (−0.9, 2.2)	1.1 (−0.5, 2.8)	144	2.2 (0.7, 3.7)**	1.8 (0.2, 3.4)*
Stayed partnered	645	REF	REF	743	REF	REF
Became separated/divorced/widowed	39	−1.3 (−3.6, 0.9)	−1.6 (−3.9, 0.7)	58	−1.0 (−2.9, 0.8)	−1.1 (−2.9, 0.8)
BMI (kg/m^2^)						
Stayed not partnered	150	REF	REF	171	REF	REF
Became partnered	174	−0.1 (−0.6, 0.5)	0.1 (−0.5, 0.6)	144	0.9 (0.3, 1.4)**	0.7 (0.1, 1.3)*
Stayed partnered	645	REF	REF	743	REF	REF
Became separated/divorced/widowed	39	−0.4 (−1.2, 0.3)	−0.5 (− 1.3, 0.3)	58	− 0.5 (− 1.2, 0.2)	−0.5 (− 1.2, 0.2)
Total PA (mins/wk)						
Stayed not partnered	56	REF	REF	123	REF	REF
Became partnered	84	101.9 (−51.6, 255.5)	175.3 (17.2, 333.4)*	78	84.5 (−41.7, 210.7)	65.5 (−65.4, 196.4)
Stayed partnered	276	REF	REF	554	REF	REF
Became separated/divorced/widowed	16	54.6 (−174.1, 283.4)	46.8 (− 184.1, 277.8)	34	67.4 (−86.8, 221.6)	82.7 (−73.3, 238.8)
Walking PA (mins/wk)						
Stayed not partnered	56	REF	REF	123	REF	REF
Became partnered	84	−2.3 (−84.0, 79.3)	43.2 (−41.0, 127.5)	78	− 20.8 (−92.0, 50.3)	10.9 (−62.5, 84.3)
Stayed partnered	276	REF	REF	554	REF	REF
Became separated/divorced/widowed	16	47.6 (−74.0, 169.3)	19.8 (−103.2, 142.9)	34	86.3 (−0.7, 173.3)	65.4 (−22.2, 153.0)
Moderate intensity PA (mins/wk)						
Stayed not partnered	56	REF	REF	123	REF	REF
Became partnered	84	88.1 (9.4, 166.8)*	86.8 (4.6, 169.0)*	78	101.9 (16.7, 187.1)*	37.0 (−49.4, 123.4)
Stayed partnered	276	REF	REF	554	REF	REF
Became separated/divorced/widowed	16	−26.7 (−144.0, 90.6)	−7.0 (−127.1, 113.1)	34	3.4 (−100.6, 107.4)	47.1 (−55.7, 150.0)
Vigorous intensity PA (mins/wk)						
Stayed not partnered	56	REF	REF	123	REF	REF
Became partnered	84	21.4 (−44.2, 86.9)	51.0 (−16.1, 118.0)	78	5.1 (−31.1, 41.3)	22.2 (−14.9, 59.3)
Stayed partnered	276	REF	REF	554	REF	REF
Became separated/divorced/widowed	16	30.4 (−67.3, 128.1)	29.2 (−68.8, 127.2)	34	−28.0 (−72.2, 16.2)	−40.3 (−84.4, 3.9)
Occupational PA (mins/wk)						
Stayed not partnered	56	REF	REF	123	REF	REF
Became partnered	84	40.9 (−70.5, 152.3)	62.0 (−52.8, 176.9)	78	6.8 (−62.9, 76.5)	45.5 (−25.0, 116.0)
Stayed partnered	276	REF	REF	554	REF	REF
Became separated/divorced/widowed	16	−84.9 (− 250.9, 81.2)	−70.9 (− 238.7, 96.9)	34	− 8.4 (−93.4, 76.6)	−46.8 (− 130.7, 37.1)
Transport PA (mins/wk)						
Stayed not partnered	56	REF	REF	123	REF	REF
Became partnered	84	14.0 (−34.0, 62.0)	32.8 (−17.2, 82.8)	78	−40.6 (−86.7, 5.4)	− 38.4 (− 86.0, 9.3)
Stayed partnered	276	REF	REF	554	REF	REF
Became separated/divorced/widowed	16	55.7 (−15.9, 127.2)	44.8 (−28.2, 117.9)	34	57.3 (1.1, 113.5)*	62.0 (5.3, 118.8)*
Domestic PA (mins/wk)						
Stayed not partnered	56	REF	REF	123	REF	REF
Became partnered	84	100.2 (37.5, 162.9)**	96.7 (30.8, 162.7)**	78	129.3 (46.4, 212.1)**	47.3 (−35.2, 129.8)
Stayed partnered	276	REF	REF	554	REF	REF
Became separated/divorced/widowed	16	−13.1 (− 106.5, 80.4)	−5.3 (− 101.7, 91.1)	34	18.5 (−82.6, 119.7)	75.7 (−22.5, 173.9)
Leisure time PA (mins/wk)						
Stayed not partnered	56	REF	REF	123	REF	REF
Became partnered	84	−45.9 (−106.8, 14.9)	−8.8 (−71.8, 54.2)	78	−5.2 (−52.8, 42.3)	19.7 (−28.9, 68.4)
Stayed partnered	276	REF	REF	554	REF	REF
Became separated/divorced/widowed	16	93.0 (2.3, 183.6)*	70.4 (−21.7, 162.4)	34	−8.8 (−66.7, 49.0)	−22.7 (− 80.6, 35.2)
Sitting time (mins/day)						
Stayed not partnered	56	REF	REF	123	REF	REF
Became partnered	84	3.7 (− 50.0, 57.3)	5.0 (−50.3, 60.3)	78	−43.3 (−83.0, −3.5)*	− 5.1 (− 44.8, 34.6)
Stayed partnered	276	REF	REF	554	REF	REF
Became separated/divorced/widowed	16	−34.3 (−114.3, 45.7)	−49.4 (− 130.4, 31.5)	34	50.0 (1.7, 98.3)*	27.8 (−19.2, 74.8)
TV viewing time (mins/day)						
Stayed not partnered	56	REF	REF	123	REF	REF
Became partnered	84	−5.6 (−34.2, 23.0)	4.5 (−25.4, 34.5)	78	−6.6 (− 24.8, 11.6)	−4.2 (− 23.1, 14.6)
Stayed partnered	276	REF	REF	554	REF	REF
Became separated/divorced/widowed	16	31.9 (−10.7, 74.6)	25.0 (−18.8, 68.8)	34	−13.3 (−35.6, 9.0)	− 15.9 (− 38.4, 6.5)

BMI, body mass index; CI, confidence interval; PA, physical activity

Model 1: adjusted for weight, PA or sedentary behaviours at baseline

Model 2: Model 1 + baseline age, sex, education, follow-up length and parenting transitions

β (95% CI) in bold means statistically significant difference compared to the reference group (became partnered vs. stayed not partnered, became separated/divorced/widowed vs. stayed partnered both times)

*P ≤ 0.05, **P ≤ 0.01

Total PA decreased among all partnering transition groups except for men and women who became separated/divorced/widowed and women who stayed partnered (Table S2). Compared to men who stayed not partnered, men who became partnered reported higher levels of total, moderate intensity and domestic PA at follow-up. Partnering transitions had little effect on women’s total and domain-specific PA at follow-up. There were no significant differences in sedentary behaviours at follow-up across partnering transition groups among men or women.

Weight increased among all parental groups over the 5-year follow-up (Table S3). Relative to staying child-free, having a first child was associated with greater weight and BMI at follow-up among women (Table 4). There was no significant difference in weight or BMI at follow-up between those who had the same number of children and those who had additional children.

**Table 4:** β (95% CI) for weight, domain-specific physical activity and sedentary behaviours by change in parental status, Childhood Determinants of Adult Health Study, Australia, 1985–2011

	Men			Women		
	n	Model 1	Model 2	n	Model 1	Model 2
		β (95% CI)	β (95% CI)		β (95% CI)	β (95% CI)
Weight (kg)						
Stayed child-free	308	REF	REF	309	REF	REF
Had first child	275	−1.0 (−2.1, 0.2)	−1.3 (− 2.7, 0)	213	1.2 (0, 2.4)*	1.4 (0.1, 2.7)*
Same number of children	229	REF	REF	382	REF	REF
Had additional children	196	−0.7 (−2.1, 0.6)	−0.8 (− 2.2, 0.6)	212	−1.0 (− 2.2, 0.1)	−0.8 (− 1.9, 0.4)
BMI (kg/m^2^)						
Stayed child-free	308	REF	REF	309	REF	REF
Had first child	275	−0.3 (−0.7, 0)	− 0.4 (− 0.8, 0.1)	213	0.5 (0, 1.0)*	0.5 (0, 1.0)
Same number of children	229	REF	REF	382	REF	REF
Had additional children	196	−0.3 (−0.8, 0.1)	−0.3 (− 0.8, 0.2)	212	−0.4 (− 0.9, 0)	−0.3 (− 0.8, 0.1)
Total PA (mins/wk)						
Stayed child-free	145	REF	REF	213	REF	REF
Had first child	126	− 115.4 (−222.8, −7.9)*	−123.9 (− 248.8, 1.1)	145	87.5 (−6.9, 182.0)	36.2 (−68.6, 141.1)
Same number of children	80	REF	REF	245	REF	REF
Had additional children	81	119.7 (−20.1, 259.5)	126.1 (−15.1, 267.4)	186	50.4 (−35.5, 136.3)	56.9 (−31.7, 145.4)
Walking PA (mins/wk)						
Stayed child-free	145	REF	REF	213	REF	REF
Had first child	126	−95.6 (−152.9, −38.3)***	−76.1 (− 142.6, −9.6)*	145	−85.3 (− 137.9, − 32.7)**	−83.1 (− 141.8, −24.4)**
Same number of children	80	REF	REF	245	REF	REF
Had additional children	81	17.6 (−57.3, 92.5)	35.4 (−40.3, 111.1)	186	−48.9 (−96.4, −1.3)*	−37.6 (−86.9, 11.7)
Moderate intensity PA (mins/wk)						
Stayed child-free	145	REF	REF	213	REF	REF
Had first child	126	33.3 (−22.0, 88.7)	29.9 (−35.0, 94.8)	145	204.3 (142.1, 266.5)***	175.0 (105.9, 244.1)***
Same number of children	80	REF	REF	245	REF	REF
Had additional children	81	71.4 (−0.2, 143.1)	80.0 (6.7, 153.3)	186	102.1 (45.4, 158.8)***	107.2 (48.8, 165.6)***
Vigorous intensity PA (mins/wk)						
Stayed child-free	145	REF	REF	213	REF	REF
Had first child	126	−48.0 (−94.0, −2.0)*	−68.2 (− 121.4, −15.0)*	145	−42.3 (−69.1, −15.4)**	−64.7 (− 94.4, −34.9)***
Same number of children	80	REF	REF	245	REF	REF
Had additional children	81	15.0 (−44.4, 74.3)	3.2 (−56.7, 63.0)	186	−13.4 (−37.7, 10.9)	−23.8 (− 48.8, 1.2)
Occupational PA (mins/wk)						
Stayed child-free	145	REF	REF	213	REF	REF
Had first child	126	−23.9 (− 102.1, 54.2)	−1.7 (−92.4, 89.0)	145	−76.5 (− 127.3, − 25.7)**	−105.7 (− 162.0, −49.4)***
Same number of children	80	REF	REF	245	REF	REF
Had additional children	81	72.5 (−28.9, 173.9)	87.5 (−14.9, 190.0)	186	−115.2 (− 161.2, −69.2)***	−115.6 (− 163.0, − 68.3)***
Transport PA (mins/wk)						
Stayed child-free	145	REF	REF	213	REF	REF
Had first child	126	−38.9 (−72.6, −5.1)*	−50.8 (−90.3, − 11.3)*	145	−14.7 (−49.0, 19.5)	− 1.7 (− 39.8, 36.4)
Same number of children	80	REF	REF	245	REF	REF
Had additional children	81	−0.3 (−44.1, 43.5)	−8.5 (−53.3, 36.2)	186	28.1 (−2.8, 59.0)	36.5 (4.4, 68.5)*
Domestic PA (mins/wk)						
Stayed child-free	145	REF	REF	213	REF	REF
Had first child	126	33.7 (−10.9, 78.3)	25.7 (−26.5, 77.8)	145	234.9 (175.6, 294.2)***	221.6 (155.6, 287.5)***
Same number of children	80	REF	REF	245	REF	REF
Had additional children	81	22.7 (−35.0, 80.4)	29.1 (−29.8, 87.9)	186	139.0 (85.1, 192.9)***	147.4 (91.7, 203.0)***
Leisure time PA (LTPA, mins/wk)^a^						
Stayed child-free	145	REF	REF	202	REF	REF
Had first child	126	−82.5 (− 125.0, −40.0)***	−89.7 (− 139.6, −39.9)***	126	−64.7 (−102.0, −27.4)***	−84.7 (− 125.7, −43.6)***
Same number of children	80	REF	REF	236	REF	REF
Had additional children	81	3.6 (−51.4, 58.6)	3.7 (−52.6, 59.9)	145	−14.7 (−49.3, 19.9)	−25.7 (−61.0, 9.6)
Sitting time (mins/day)^b^						
Stayed child-free	141	REF	REF	213	REF	REF
Had first child	124	−16.2 (−54.2, 21.9)	−34.7 (−79.1, 9.7)	145	−117.0 (− 145.5, −88.6)***	−103.4 (− 135.0, − 71.8)***
Same number of children	78	REF	REF	245	REF	REF
Had additional children	76	5.1 (−44.8, 55.0)	−10.6 (−61.2, 39.9)	186	−42.8 (− 68.5, − 17.0)***	−39.3 (− 65.9, − 12.8)**
TV viewing time (mins/day)^a^						
Stayed child-free	145	REF	REF	202	REF	REF
Had first child	126	−25.5 (−45.5, −5.4)*	−26.4 (−49.9, − 2.8)*	126	−11.2 (− 25.9, 3.4)	−8.0 (− 24.3, 8.2)
Same number of children	80	REF	REF	236	REF	REF
Had additional children	81	−1.7 (−27.6, 24.3)	0.6 (−26.0, 27.3)	145	−3.1 (− 16.7, 10.5)	−1.7 (− 15.6, 12.3)

BMI, body mass index; CI, confidence interval; PA, physical activity

Model 1: adjusted for weight, PA or sedentary behaviours at baseline

Model 2: Model 1 + baseline age, sex, education, follow-up length and marital transitions

^a^Model 2 + baseline diet quality in women

^b^Model 2 + baseline diet quality in men

β (95% CI) in bold means statistically significant difference compared to the reference group (had first child born vs. stayed child-free, had additional children vs. same number of children)

*P ≤ 0.05, **P ≤ 0.01, ***P ≤ 0.001

Among men, those who had their first child during follow-up reported 123.9 min/week less total PA at follow-up than those who remained child-free (*P* = 0.052), independent of baseline value and other confounders (Table 4). They spent less time in walking, vigorous intensity, transport, and leisure time PA at follow-up.

During the 5-year follow-up, time spent sitting and watching TV decreased among all parental groups except the group remaining child-free (Table S3). Relative to staying child-free, transition into parenthood with a first child was associated with 26.4 min/day less TV viewing time at follow-up among men and 103.4 min/day less sitting time at follow-up among women. For women only, having additional children was associated with 39.3 min/day less sitting time at follow-up than having the same number of children.

In sensitivity analyses to examine the effects of loss to follow-up on results, similar findings to those reported in Tables 3 and 4 were observed when applying combined MI and IPW (data not shown). The results obtained after excluding women who had their baby in the last 3 months were broadly similar to the original results in direction and magnitude (the change in β coefficient ranged from − 10.6 to 43.5%, Table S4), but the statistically significant greater weight gain for women having their first child relative to those staying child-free was no longer apparent (β, 95% CI: 0.8, − 0.5 to 2.1).

## Discussion

In this longitudinal study of Australian adults, we found that the associations of partnering and parenting transitions with changes in weight, domain-specific PA and sedentary behaviours differed between men and women. Compared to remaining not partnered, partnering was associated with an increase in total PA among men and a greater weight gain among women. Men who had their first child reported greater reductions in total PA and TV viewing time than those remaining child-free. Women having a first child had greater weight gain but spent less time sitting than those remaining child-free. Having additional children was associated with less sitting time than having the same number of children among women.

We found that partnering and parenting transitions were differentially associated with changes in weight among men and women. Greater weight gain was observed among women who became partnered than those who stayed not partnered and among women who had first child than those who stayed child-free, while no significant differences were observed among men. These results are supported by some longitudinal studies [20, 34] but not others [5, 16]. The attractiveness model may help to explain the greater weight gain associated with transition to partnership in women only. In a survey of preferred traits in a relationship partner, physical attractiveness was rated as being more important by men than women [35] and weight is one of the important physical attractiveness characteristics. Partnered people may be less likely to be concerned about their weight because they are not in marriage market [36]. Another possible reason for the sex difference is that, as we found, men who became partnered tended to be more active compared to their counterparts who stayed unpartnered while there was no significant difference between these two groups among women. Excluding women who recently had a baby took away the statistically significant greater weight gain among those who had their first child than those who stayed child-free, suggesting a short-term weight gain after giving birth.

The benefits of partnering on PA levels were greater for men than women. Men who became partnered showed higher total PA at follow-up than those who stayed not partnered. They tended to increase their domestic PA and moderate intensity PA during follow-up. Partnering transitions appeared to have little effect on women’s PA though women who became separated/divorced/widowed had higher transport PA at follow-up than their peers who remained partnered. To the authors’ knowledge, this is the first longitudinal report of change in total PA by partnering transitions among men. Several studies of similar design among men all focused on leisure time PA [16–19, 37, 38] and only one of them examined participants in their 20s when partnering often takes place [18]. Our finding of no significant difference in men’s leisure time PA by partnering transitions is supported by this comparable study [18]. The greater PA benefits of partnership to men than women may be partly explained by gender and marital roles. Compared to men, women are generally more knowledgeable of health-related issues and are more likely to assume responsibility for health of their partners and encourage them to conform to health norms [19, 39], such as being more active.

Compared to men who stayed child-free, men who had their first child decreased their total PA (*P* = 0.052). They tended to decrease their walking, vigorous intensity, transport and leisure time PA more than those who remained child-free. Although no significant difference in total PA at follow-up was observed between these two groups among women, there were both healthy and unhealthy changes in PA by intensity and domain as detailed in results. Only one previous study has attempted to explore how sex moderates the longitudinal relationship between parenting transitions and PA, but that study only assessed leisure time PA [18]. Our finding in men is supported by this study but not the results in women [18]. The inconsistent finding in women may be because that study only had a small number of women who had their first child (*n* = 16) and included a high proportion of pregnant women at baseline [22]. It is well documented that pregnancy is a period of decreased PA [40].

Relative to having the same number of children, having additional children had little effect on men’s PA but was associated with both increases and reductions in women’s PA by intensity and domain, despite no significant change in total PA. These results are consistent with prior evidence in men [18] and women [12, 14, 18] but all previous studies only assessed leisure time PA. Time constraints and being out of the work force may help to explain the significant reduction in occupational PA and increases in domestic and moderate intensity PA among women compared with men. This speculation was supported by Australian and international research. In a large-scale, longitudinal and nationally representative study of children and families from Australia, highly gendered patterns in the time-use distribution of parents of young children were found. Fathers spent more time than mothers in paid employment, but less time caring for children and domestic work [41]. Similar results were reported in studies from other countries [42]. Even when both parents undertook full-time paid work, mothers continued to do more child care and house work [43]. Having additional children can further change women’s time and energy commitments, such as spending less time in work and leisure time PA and more time in domestic PA, as seen in this study.

Having a first child was associated with less sitting time in women and less TV viewing time in men than remaining child-free. The finding is consistent with previous cross-sectional studies conducted in Australia [44] and Canada [45], which showed an inverse relationship between number of children and sitting time. This might be simply because child care limits time for relaxation and entertainment. Another possible explanation is change in employment status (e.g. taking maternity leave and returning to work part time). Hours worked per week have been associated with greater sitting time, with women working full time spending more time sitting [44]. Alternatively, parents may decrease their sedentary behaviours to create a health-promoting environment for their young children [46]. Having additional children was also found to be associated with less sitting time than having the same number of children among women. This might be explained by women’s further time constraints and being out of the work force.

No significant differences were observed between baseline and follow-up levels of total, vigorous intensity, transport, leisure time and walking PA, and TV viewing time. Our results concur with prior evidence that suggests that mid-adulthood is a period where these PA and sedentary behaviour plateau [47, 48] but not all [49]. For example, using data from a nationally representative sample of adults from the US (*n* = 43,732), Caspersen et al. [47] showed that leisure time PA patterns (i.e. prevalence of physical inactivity; regular, sustained PA; and regular, vigorous PA) in mid-adulthood (30–64 years) were relatively stable and the absolute annual rates of change in all aforementioned leisure time PA patterns were less than 0.5 percentage points per year. In contrast to Caspersen and colleagues’ findings, using accelerometer-measured PA in a nationally representative health survey in US, Troiano et al. [49] concluded that total PA declined with age across the lifespan although the declines in mid-adulthood were at a slower rate than in adolescence. Possible explanations for the discrepancies may include differences in PA measurements (i.e. self-reported vs. objective-measured) and types (i.e. total vs. leisure time PA), definitions of age groups (i.e. 5-year vs. 10-year) and study designs (i.e. cross-sectional vs. longitudinal).

We did not find a significant difference in sedentary behaviours at follow-up between those who experienced partnering transitions and those who did not, suggesting partnering and partnership termination do not play a role in predicting future sedentary behaviours. TV viewing time was similar between baseline and follow-up in both men and women, averaging about 2 h daily. This amount of time was about half of that reported from the 2006 Australian Time Use Surveys [50]. Our participants’ higher education level (bachelor’s degree or higher 42.2% vs. 22.1% in the general population of the same age group) and lower proportion not in labour force (10.8% vs. 32.9%) might help to explain the difference. Although the evidence is not conclusive [51], most studies support a link between low educational attainment or not being in paid work and prolonged TV viewing time (≥2 h/day) [52, 53]. Growing use of other digital media (e.g. smartphone, tablet and laptop) in the period of follow-up through to 2009–11 may have contributed to changes in TV watching, but the impact on the results is likely minimal since there is no evidence that TV watching evolves differently by marital or parental status.

Some limitations should be acknowledged when interpreting the results. First, self-reported weight might underestimate the actual weight. However, we applied a correction factor to reduce the potential error, and the outcome of interest calculation – weight change based on self-reported weight from baseline to follow-up has been identified as a valid estimate with minimal error [54]. Second, there might be measurement error and recall bias in PA and sedentary behaviours as these data were collected by means of self-completed questionnaires and participants were asked to recall their PA for each domain over the past week. PA from IPAQ-L has a tendency towards over-reporting [55, 56] and time spent in sedentary behaviours is prone to under-reporting [57]. However, these measurements have been validated in a number of studies and are widely accepted in the literature. Although lower than device-measured sitting time (varying greatly in the literature, mostly over 9 h/day) [57], our estimate of sitting time (5–6 h/day) is similar to other reports from the Australian general population of similar age in 2011(males 6.4 h/day and females 5.3 h/day) [58] and some studies which also used the IPAQ-L to measure sitting time (about 6 h/day) [56, 59]. There is no evidence that the accuracy of self-reported PA and sedentary behaviours differs by marital and parental status. Most importantly, our outcome of interest is change in these behaviours, not data at one time point. To the extent that the errors of measurement at different time points are correlated, calculating changes in PA and sedentary behaviours should help to reduce their impact on the estimated associations. In addition, IPAQ-L only reports absolute PA time. For example, total working time is not taken into account in the calculation of occupational PA (same for other PA domains). Therefore, the observed large changes in PA over time could be simply due to changes in participants daily lives (e.g. becoming partnered or transition into parenthood). Third, only biological children were included in the definition of parental status. There might be misclassification of participants who adopted children or who became a step-parent. Fourth, the variable of partnering transitions was defined based on marital status at two-time points, 5-years apart. It is possible that other marital transitions have occurred during the follow-up. Besides, the increase in total PA among men and weight among women who became partnered might have been underestimated, since participants who were in a relationship but living apart were classified as single. This may have diluted the associations of partnering transitions with changes in PA and weight. However, our definition of marital status is widely used in Australian national reports [60] and the literature [61], and the proportion of Australian adults falling into this category is low (9%) [62]. Fifth, the sample size for some sub analyses is rather small. Future larger studies are needed to confirm our findings. Last, up to two-thirds of participants were not included in the analyses of changing PA and sedentary behaviours for various reasons (e.g. loss to follow-up, being pregnant or missing information on covariates). Comparison of socio-demographic characteristics between those included and those not included revealed that participants included were more likely to be younger, female, more highly educated and employed as professionals or managers, married or living as married and had children at baseline. However, we applied combined MI and IPW to account for these differences and found the original results were largely unchanged.

The strengths of this study include its large national sample, the prospective design, the mutual adjustment for partnering and parenting transitions in analyses, the sex stratified analyses and the use of advanced methodology to account for loss to follow-up. In addition, this is the first study to examine the longitudinal relationships between two important life stage transitions in young adulthood and domain-specific PA and sedentary behaviours. This is important as these results could help to understand the domain-specific changes in PA and thereby illuminate the most opportune interventions to help mitigate the declined PA during parenthood.

## Conclusions

Partnering was associated with increases in men’s total PA and women’s weight. Transitions into parenthood with a first child or additional children were associated with potentially health-impairing changes in weight and PA, but health-promoting changes in sedentary behaviours among men and women. Although the precise mechanisms through which greater weight gain occurs among women are likely to be complex and remain uncertain, our findings add to a growing body of evidence that women’s weight is more easily influenced by partnering and parenting transitions than men’s, and thus deserves specific attention in relation to obesity prevention. This study identified an important high-risk group for targeting of PA strategies – men who had their first child. To mitigate their decreased total PA, our domain-specific results suggest that future promotion strategies should focus on increasing transport and leisure time PA. Future research with objectively measured PA and sedentary time (e.g. through accelerometry) and larger samples are needed to verify our findings.

## Supplementary information

**Additional file 1: Table S1.** Childhood characteristics of participants assessed and not assessed at baseline in adulthood, Childhood Determinants of Adult Health Study, Australia, 1985-2011^a^. **Table S2.** Mean and standard deviation for weight, domain-specific physical activity and sedentary behaviours at baseline and follow-up by change in marital status, Childhood Determinants of Adult Health Study, Australia, 1985–2011. **Table S3.** Mean and standard deviation for weight, domain-specific physical activity and sedentary behaviours at baseline and follow-up by change in parental status, Childhood Determinants of Adult Health Study, Australia, 1985–2011. **Table S4.** β (95% CI) for weight by change in parental status before or after removing women who had their baby in the last 3 months, Childhood Determinants of Adult Health Study, Australia, 1985–2011.

## Data Availability

The datasets used and/or analysed during the current study are available from the corresponding author on reasonable request.

## Abbreviations

ASHFS: Australian Schools Health and Fitness Survey
BMI: Body Mass Index
CDAH: Childhood Determinants of Adult Health
CI: Confidence Interval
IPAQ: International Physical Activity Questionnaire
IPW: Inverse Probability Weighting
METs: Metabolic Equivalents
MI: Multiple Imputation
PA: Physical Activity
SD: Standard Deviation
TV: Television
US: United States
