# Supplementary material for: Partnering and parenting transitions in Australian men and women: associations with changes in weight, domain-specific physical activity and sedentary behaviours
Source: Int J Behav Nutr Phys Act. 2020 Jul 8;17:87. doi: 10.1186/s12966-020-00989-6 (PMC7346521; doi:10.1186/s12966-020-00989-6)
Supplement: Supplementary file 1 — Additional file 1: Table S1. Childhood characteristics of participants assessed and not assessed at baseline in adulthood, Childhood Determinants of Adult Health Study, Australia, 1985-2011a. Table S2. Mean and standard deviation for weight, domain-specific physical activity and sedentary behaviours at baseline and follow-up by change in marital status, Childhood Determinants of Adult Health Study, Australia, 1985–2011. Table S3. Mean and standard deviation for weight, domain-specific physical activity and sedentary behaviours at baseline and follow-up by change in parental status, Childhood Determinants of Adult Health Study, Australia, 1985–2011. Table S4. β (95% CI) for weight by change in parental status before or after removing women who had their baby in the last 3 months, Childhood Determinants of Adult Health Study, Australia, 1985–2011. [file 12966_2020_989_MOESM1_ESM.docx]

**Table S1** Childhood characteristics of participants assessed and not assessed at baseline in adulthood, Childhood Determinants of Adult Health Study, Australia, 1985-2011^a^

| **Characteristics** | **Not assessed (n=4,523)** | **Assessed (n=3,975)** | **P-value** |
| --- | --- | --- | --- |
| Age (years), Mean (SD) | 10.8 (2.6) | 11.1 (2.5) | <0.001 |
| Male, % (n) | 55.0 (2487) | 45.8 (1820) | <0.001 |
| Area-level disadvantage, % (n) |  |  | <0.001 |
| High | 22.6 (739) | 24.8 (751) |  |
| Medium-high | 29.4 (962) | 27.7 (838) |  |
| Medium-low | 37.1 (1,214) | 40.1 (1,213) |  |
| Low | 10.9 (356) | 7.5 (226) |  |
| Weight status, % (n) |  |  | <0.001 |
| Normal (BMI<25) | 87.0 (3929) | 89.7 (3562) |  |
| Overweight (BMI≥25 to 29.9) | 11.1 (500) | 9.1 (361) |  |
| Obese (BMI≥30) | 2.0 (89) | 1.3 (50) |  |
| Self-rated health, % (n) |  |  | 0.026 |
| Very good/good | 77.8 (2,582) | 80.4 (2,484) |  |
| Average | 20.7 (686) | 18.4 (568) |  |
| Poor/very poor | 1.5 (51) | 1.2 (36) |  |
| Number of sit-ups in 5 minutes, Mean (SD) | 36.6 (29.3) | 40.5 (29.8) | <0.001 |
| Standing long jump (cm), Mean (SD) | 141.8 (28.9) | 144.7 (29.0) | <0.001 |
| Number of push-ups in 30 seconds, Mean (SD) | 10.7 (6.5) | 10.4 (6.6) | 0.050 |
| Sit and reach (cm), Mean (SD) | 3.7 (6.8) | 4.7 (7.2) | <0.001 |
| Height (cm), Mean (SD) | 144.9 (15.4) | 146.8 (15.4) | <0.001 |
| Weight (kg), Mean (SD) | 39.5 (13.0) | 40.3 (13.0) | 0.007 |
| Arm girth (cm), Mean (SD) | 22.2 (3.4) | 22.2 (3.3) | 0.473 |
| Waist girth (cm), Mean (SD) | 63.7 (8.8) | 63.5 (8.4) | 0.376 |
| Hip girth (cm), Mean (SD) | 75.9 (10.3) | 76.6 (10.2) | 0.004 |

BMI: body mass index; SD: standard deviation.

^a^ Sample size varied because of missing data (range, 3,271-4,521 for not assessed group and 3,028-3,974 for assessed group).

**Table S2** Mean and standard deviation for weight, domain-specific physical activity and sedentary behaviours at baseline and follow-up by change in marital status, Childhood Determinants of Adult Health Study, Australia, 1985-2011

| **Variables** | **Men** | | | | | |  | **Women** | | | | | |
| --- | --- | --- | --- | --- | --- | --- | --- | --- | --- | --- | --- | --- | --- |
|  | **n** | **Baseline** |  | **Follow-up** |  | **Change** |  | **n** | **Baseline** |  | **Follow-up** |  | **Change** |
|  |  | **Mean (SD)** |  | **Mean (SD)** |  | **Mean (SD)** |  |  | **Mean (SD)** |  | **Mean (SD)** |  | **Mean (SD)** |
| Weight (kg) |  |  |  |  |  |  |  |  |  |  |  |  |  |
| Stayed not partnered | 150 | 84.1 (16.4) |  | 86.8 (16.7) |  | 2.6 (6.4) |  | 171 | 70.8 (17.0) |  | 73.0 (17.8) |  | 2.3 (6.7) |
| Became partnered | 174 | 83.7 (13.9) |  | 87.0 (15.1) |  | 3.3 (7.1) |  | 144 | 65.7 (13.6) |  | 70.3 (15.8) |  | 4.6 (7.8) |
| Stayed partnered | 645 | 86.0 (14.0) |  | 88.3 (14.9) |  | 2.4 (7.3) |  | 743 | 69.1 (14.4) |  | 71.3 (15.3) |  | 2.3 (6.7) |
| Became separated/divorced/widowed | 39 | 86.2 (15.4) |  | 87.3 (15.1) |  | 1.0 (6.0) |  | 58 | 68.9 (12.4) |  | 70.1 (13.7) |  | 1.3 (7.1) |
| BMI (kg/m^2^) |  |  |  |  |  |  |  |  |  |  |  |  |  |
| Stayed not partnered | 150 | 26.2 (4.8) |  | 27.2 (5.7) |  | 1.0 (2.4) |  | 171 | 25.8 (5.9) |  | 26.4 (6.2) |  | 0.6 (2.6) |
| Became partnered | 174 | 25.9 (4.2) |  | 26.8 (5.1) |  | 0.9 (2.8) |  | 144 | 24.0 (4.8) |  | 25.5 (5.9) |  | 1.5 (3.1) |
| Stayed partnered | 645 | 26.6 (3.9) |  | 27.3 (4.4) |  | 0.8 (2.3) |  | 743 | 25.0 (5.0) |  | 25.7 (5.5) |  | 0.7 (2.6) |
| Became separated/divorced/widowed | 39 | 26.3 (3.9) |  | 26.6 (3.9) |  | 0.3 (2.0) |  | 58 | 24.6 (4.5) |  | 24.9 (5.0) |  | 0.2 (2.8) |
| Total PA (mins/wk) |  |  |  |  |  |  |  |  |  |  |  |  |  |
| Stayed not partnered | 56 | 733.6 (609.4) |  | 648.0 (514.7) |  | -85.6 (569.4) |  | 123 | 727.6 (516.1) |  | 602.2 (396.6) |  | -126.6 (547.7) |
| Became partnered | 84 | 791.5 (553.3) |  | 774.0 (546.7) |  | -17.4 (576.5) |  | 78 | 722.9 (459.2) |  | 685.1 (389.2) |  | -33.6 (468.0) |
| Stayed partnered | 276 | 724.7 (507.5) |  | 686.0 (493.1) |  | -38.7 (540.0) |  | 554 | 702.6 (457.5) |  | 776.2 (494.0) |  | 74.0 (548.0) |
| Became separated/divorced/widowed | 16 | 703.1 (519.7) |  | 731.6 (381.4) |  | 28.5 (441.9) |  | 34 | 594.5 (338.6) |  | 805.9 (530.7) |  | 211.4 (473.5) |
| Walking PA (mins/wk) |  |  |  |  |  |  |  |  |  |  |  |  |  |
| Stayed not partnered | 56 | 283.8 (317.5) |  | 289.0 (291.8) |  | 5.2 (366.8) |  | 123 | 303.2 (274.6) |  | 286.9 (269.8) |  | -16.3 (310.4) |
| Became partnered | 84 | 315.9 (354.4) |  | 297.6 (298.7) |  | -18.3 (360.8) |  | 78 | 291.2 (261.9) |  | 262.7 (237.1) |  | -28.5 (326.1) |
| Stayed partnered | 276 | 242.9 (242.4) |  | 208.1 (235.3) |  | -34.8 (269.0) |  | 554 | 231.7 (236.0) |  | 227.6 (257.2) |  | -4.1 (303.1) |
| Became separated/divorced/widowed | 16 | 256.5 (314.7) |  | 260.4 (295.8) |  | 3.9 (287.9) |  | 34 | 154.1 (133.3) |  | 292.6 (291.6) |  | 138.5 (274.6) |
| Moderate intensity PA (mins/wk) |  |  |  |  |  |  |  |  |  |  |  |  |  |
| Stayed not partnered | 56 | 275.8 (196.7) |  | 229.2 (207.5) |  | -46.7 (257.1) |  | 123 | 306.0 (292.9) |  | 240.5 (231.2) |  | -65.5 (296.3) |
| Became partnered | 84 | 301.0 (273.6) |  | 325.1 (276.8) |  | 24.1 (345.9) |  | 78 | 299.9 (246.5) |  | 340.3 (288.0) |  | 40.5 (349.3) |
| Stayed partnered | 276 | 310.4 (235.6) |  | 317.9 (240.0) |  | 7.5 (268.4) |  | 554 | 403.9 (326.4) |  | 462.9 (335.1) |  | 59.0 (380.6) |
| Became separated/divorced/widowed | 16 | 291.8 (291.0) |  | 285.5 (234.5) |  | -6.4 (308.6) |  | 34 | 371.5 (300.0) |  | 455.3 (359.2) |  | 83.9 (348.9) |
| Vigorous intensity PA (mins/wk) |  |  |  |  |  |  |  |  |  |  |  |  |  |
| Stayed not partnered | 56 | 174.0 (281.3) |  | 129.9 (187.1) |  | -44.1 (257.1) |  | 123 | 118.5 (217.2) |  | 74.9 (106.3) |  | -43.6 (219.7) |
| Became partnered | 84 | 174.5 (227.8) |  | 151.4 (207.3) |  | -23.2 (225.0) |  | 78 | 131.8 (182.1) |  | 82.0 (146.4) |  | -49.8 (212.3) |
| Stayed partnered | 276 | 171.5 (210.4) |  | 160.0 (207.2) |  | -11.5 (258.3) |  | 554 | 67.1 (134.7) |  | 85.7 (132.9) |  | 18.6 (171.7) |
| Became separated/divorced/widowed | 16 | 154.7 (172.9) |  | 185.8 (133.8) |  | 31.0 (247.6) |  | 34 | 69.0 (85.0) |  | 57.9 (105.4) |  | -11.0 (110.3) |
| Occupational PA (mins/wk) |  |  |  |  |  |  |  |  |  |  |  |  |  |
| Stayed not partnered | 56 | 269.7 (337.2) |  | 222.1 (314.7) |  | -47.6 (412.7) |  | 123 | 193.7 (340.4) |  | 117.8 (229.8) |  | -75.9 (379.7) |
| Became partnered | 84 | 265.9 (369.9) |  | 261.7 (366.2) |  | -4.2 (361.5) |  | 78 | 255.0 (399.9) |  | 138.1 (244.4) |  | -116.9 (396.2) |
| Stayed partnered | 276 | 294.3 (415.3) |  | 244.3 (364.9) |  | -50.0 (433.6) |  | 554 | 125.1 (261.0) |  | 128.6 (258.0) |  | 3.5 (315.5) |
| Became separated/divorced/widowed | 16 | 287.7 (369.9) |  | 157.1 (275.8) |  | -130.5 (404.1) |  | 34 | 69.4 (145.6) |  | 107.9 (267.1) |  | 38.5 (226.9) |
| Transport PA (mins/wk) |  |  |  |  |  |  |  |  |  |  |  |  |  |
| Stayed not partnered | 56 | 119.9 (182.1) |  | 105.8 (152.7) |  | -14.2 (221.5) |  | 123 | 128.1 (164.9) |  | 133.9 (173.0) |  | 5.8 (197.7) |
| Became partnered | 84 | 134.4 (207.0) |  | 122.8 (181.8) |  | -11.5 (240.1) |  | 78 | 123.3 (143.3) |  | 92.1 (104.0) |  | -31.2 (146.5) |
| Stayed partnered | 276 | 101.1 (146.8) |  | 93.9 (130.5) |  | -7.2 (168.5) |  | 554 | 99.4 (151.0) |  | 95.4 (165.2) |  | -3.9 (203.0) |
| Became separated/divorced/widowed | 16 | 86.8 (102.4) |  | 146.5 (159.5) |  | 59.7 (148.8) |  | 34 | 81.1 (82.1) |  | 148.4 (246.4) |  | 67.3 (226.0) |
| Domestic PA (mins/wk) |  |  |  |  |  |  |  |  |  |  |  |  |  |
| Stayed not partnered | 56 | 140.6 (149.0) |  | 114.7 (143.0) |  | -25.9 (192.0) |  | 123 | 198.1 (262.5) |  | 172.1 (218.5) |  | -26.0 (236.4) |
| Became partnered | 84 | 160.8 (189.6) |  | 221.1 (245.5) |  | 60.3 (257.9) |  | 78 | 184.3 (194.5) |  | 296.4 (281.0) |  | 112.1 (297.3) |
| Stayed partnered | 276 | 165.7 (166.7) |  | 194.0 (181.7) |  | 28.3 (211.6) |  | 554 | 341.4 (320.9) |  | 395.4 (331.3) |  | 54.0 (376.6) |
| Became separated/divorced/widowed | 16 | 141.7 (129.2) |  | 173.5 (187.6) |  | 31.8 (190.0) |  | 34 | 310.5 (297.8) |  | 402.7 (323.1) |  | 92.2 (326.2) |
| Leisure time PA (mins/wk) |  |  |  |  |  |  |  |  |  |  |  |  |  |
| Stayed not partnered | 56 | 203.4 (301.8) |  | 205.4 (235.3) |  | 2.1 (286.0) |  | 123 | 207.7 (240.9) |  | 178.4 (180.3) |  | -29.2 (259.6) |
| Became partnered | 84 | 230.4 (295.1) |  | 168.4 (198.0) |  | -62.0 (239.1) |  | 78 | 160.2 (191.0) |  | 158.5 (191.4) |  | -1.7 (188.6) |
| Stayed partnered | 276 | 163.7 (193.1) |  | 153.8 (187.6) |  | -9.8 (224.4) |  | 554 | 136.7 (157.3) |  | 156.8 (172.5) |  | 20.0 (198.5) |
| Became separated/divorced/widowed | 16 | 186.9 (170.0) |  | 254.5 (127.5) |  | 67.5 (247.0) |  | 34 | 133.6 (151.2) |  | 146.9 (166.1) |  | 13.4 (144.1) |
| Sitting time (mins/day) |  |  |  |  |  |  |  |  |  |  |  |  |  |
| Stayed not partnered | 56 | 386.6 (178.0) |  | 385.2 (185.7) |  | -1.4 (205.0) |  | 123 | 382.4 (170.3) |  | 393.9 (150.9) |  | 11.5 (164.1) |
| Became partnered | 84 | 370.4 (182.4) |  | 380.1 (180.0) |  | 9.8 (156.8) |  | 78 | 320.0 (163.0) |  | 326.1 (152.7) |  | 6.1 (176.0) |
| Stayed partnered | 276 | 363.6 (179.6) |  | 356.3 (188.1) |  | -7.2 (179.1) |  | 554 | 314.2 (159.7) |  | 270.1 (151.7) |  | -44.1 (171.7) |
| Became separated/divorced/widowed | 16 | 417.3 (186.6) |  | 350.9 (155.2) |  | -66.4 (177.6) |  | 34 | 328.4 (138.8) |  | 325.7 (177.4) |  | -2.8 (147.6) |
| TV viewing time (mins/day) |  |  |  |  |  |  |  |  |  |  |  |  |  |
| Stayed not partnered | 56 | 144.7 (98.2) |  | 144.7 (124.5) |  | 0 (117.2) |  | 123 | 101.6 (75.1) |  | 111.9 (82.0) |  | 10.2 (84.6) |
| Became partnered | 84 | 114.2 (73.8) |  | 126.2 (89.7) |  | 12.0 (85.1) |  | 78 | 95.4 (71.5) |  | 102.8 (70.8) |  | 7.4 (71.9) |
| Stayed partnered | 276 | 123.3 (92.4) |  | 123.0 (84.6) |  | -0.3 (98.7) |  | 554 | 101.4 (69.4) |  | 98.3 (67.1) |  | -3.1 (74.6) |
| Became separated/divorced/widowed | 16 | 163.5 (101.5) |  | 172.0 (101.6) |  | 8.4 (96.4) |  | 34 | 132.8 (106.1) |  | 97.2 (66.8) |  | -35.6 (110.9) |

BMI, body mass index; CI, confidence interval; PA, physical activity.

**Table S3** Mean and standard deviation for weight, domain-specific physical activity and sedentary behaviours at baseline and follow-up by change in parental status, Childhood Determinants of Adult Health Study, Australia, 1985-2011

|  | **Men** | | | | | |  | **Women** | | | | | |
| --- | --- | --- | --- | --- | --- | --- | --- | --- | --- | --- | --- | --- | --- |
|  | **n** | **Baseline** |  | **Follow-up** |  | **Change** |  | **n** | **Baseline** |  | **Follow-up** |  | **Change** |
|  |  | **Mean (SD)** |  | **Mean (SD)** |  | **Mean (SD)** |  |  | **Mean (SD)** |  | **Mean (SD)** |  | **Mean (SD)** |
| Weight (kg) |  |  |  |  |  |  |  |  |  |  |  |  |  |
| Stayed child-free | 308 | 83.9 (15.1) |  | 87.0 (15.4) |  | 3.1 (6.8) |  | 309 | 69.4 (16.7) |  | 71.7 (17.7) |  | 2.3 (6.7) |
| Had first child | 275 | 84.1 (12.6) |  | 86.3 (13.3) |  | 2.1 (5.6) |  | 213 | 65.3 (11.5) |  | 68.9 (14.4) |  | 3.7 (7.5) |
| Same number of children | 229 | 87.8 (16.1) |  | 90.5 (16.0) |  | 2.7 (6.4) |  | 382 | 70.4 (15.1) |  | 73.0 (15.6) |  | 2.6 (6.6) |
| Had additional children | 196 | 86.2 (13.2) |  | 88.3 (16.2) |  | 2.1 (9.8) |  | 212 | 69.1 (13.0) |  | 70.7 (13.8) |  | 1.6 (7.2) |
| BMI (kg/m^2^) |  |  |  |  |  |  |  |  |  |  |  |  |  |
| Stayed child-free | 308 | 26.0 (4.6) |  | 27.0 (5.3) |  | 1.0 (2.6) |  | 309 | 25.2 (5.8) |  | 25.8 (6.4) |  | 0.7 (2.6) |
| Had first child | 275 | 25.9 (3.5) |  | 26.5 (4.1) |  | 0.6 (1.8) |  | 213 | 23.8 (4.0) |  | 25.0 (5.3) |  | 1.2 (3.0) |
| Same number of children | 229 | 27.3 (4.5) |  | 28.3 (4.7) |  | 1.0 (2.2) |  | 382 | 25.6 (5.3) |  | 26.4 (5.6) |  | 0.8 (2.6) |
| Had additional children | 196 | 26.6 (3.6) |  | 27.3 (4.6) |  | 0.7 (3.0) |  | 212 | 24.8 (4.4) |  | 25.2 (4.8) |  | 0.4 (2.7) |
| Total PA (mins/wk) |  |  |  |  |  |  |  |  |  |  |  |  |  |
| Stayed child-free | 145 | 723.5 (532.9) |  | 724.2 (564.2) |  | 0.6 (597.3) |  | 213 | 701.4 (478.5) |  | 650.7 (432.9) |  | -50.7 (526.6) |
| Had first child | 126 | 750.1 (496.2) |  | 620.2 (436.8) |  | -130.0 (519.4) |  | 145 | 603.8 (409.2) |  | 704.7 (497.0) |  | 102.2 (526.6) |
| Same number of children | 80 | 830.3 (595.7) |  | 718.1 (444.3) |  | -112.2 (525.8) |  | 245 | 812.3 (491.9) |  | 802.0 (502.1) |  | -10.4 (558.9) |
| Had additional children | 81 | 654.1 (500.2) |  | 762.5 (531.4) |  | 108.4 (479.1) |  | 186 | 641.9 (416.1) |  | 793.8 (455.3) |  | 153.2 (531.0) |
| Walking PA (mins/wk) |  |  |  |  |  |  |  |  |  |  |  |  |  |
| Stayed child-free | 145 | 282.8 (311.0) |  | 299.7 (301.9) |  | 16.9 (343.9) |  | 213 | 301.3 (267.4) |  | 304.8 (288.3) |  | 3.5 (336.5) |
| Had first child | 126 | 256.0 (256.2) |  | 194.8 (214.8) |  | -61.2 (282.2) |  | 145 | 268.6 (254.3) |  | 211.0 (223.5) |  | -57.6 (285.3) |
| Same number of children | 80 | 317.6 (319.9) |  | 230.6 (232.9) |  | -87.0 (293.5) |  | 245 | 219.4 (243.8) |  | 247.0 (270.7) |  | 27.6 (321.9) |
| Had additional children | 81 | 183.8 (192.5) |  | 201.7 (256.4) |  | 17.9 (244.5) |  | 186 | 197.4 (187.6) |  | 192.4 (219.8) |  | -5.0 (258.0) |
| Moderate intensity PA (mins/wk) |  |  |  |  |  |  |  |  |  |  |  |  |  |
| Stayed child-free | 145 | 279.0 (231.4) |  | 265.3 (243.7) |  | -13.8 (308.2) |  | 213 | 272.9 (240.0) |  | 246.0 (205.2) |  | -26.9 (272.0) |
| Had first child | 126 | 289.8 (212.2) |  | 302.0 (231.7) |  | 12.2 (260.9) |  | 145 | 247.2 (215.4) |  | 441.7 (369.2) |  | 194.5 (363.5) |
| Same number of children | 80 | 357.7 (265.9) |  | 318.2 (231.2) |  | -39.5 (288.4) |  | 245 | 518.9 (342.8) |  | 462.7 (316.0) |  | -56.2 (362.8) |
| Had additional children | 81 | 314.6 (265.8) |  | 376.3 (265.7) |  | 61.8 (268.2) |  | 186 | 410.2 (337.4) |  | 528.2 (350.4) |  | 117.9 (410.8) |
| Vigorous intensity PA (mins/wk) |  |  |  |  |  |  |  |  |  |  |  |  |  |
| Stayed child-free | 145 | 161.7 (208.0) |  | 159.2 (202.3) |  | -2.5 (231.5) |  | 213 | 127.2 (205.9) |  | 99.9 (136.1) |  | -27.3 (231.5) |
| Had first child | 126 | 204.3 (260.0) |  | 123.4 (184.5) |  | -80.9 (260.5) |  | 145 | 88.0 (140.1) |  | 52.1 (93.1) |  | -35.9 (144.1) |
| Same number of children | 80 | 155.1 (209.6) |  | 169.3 (210.6) |  | 14.2 (265.3) |  | 245 | 74.0 (144.5) |  | 92.2 (150.7) |  | 18.2 (186.4) |
| Had additional children | 81 | 155.7 (191.9) |  | 184.5 (216.6) |  | 28.7 (240.8) |  | 186 | 34.3 (83.3) |  | 73.2 (110.1) |  | 38.9 (130.2) |
| Occupational PA (mins/wk) |  |  |  |  |  |  |  |  |  |  |  |  |  |
| Stayed child-free | 145 | 268.7 (346.6) |  | 220.2 (339.6) |  | -48.6 (409.1) |  | 213 | 207.9 (345.4) |  | 156.7 (286.9) |  | -51.3 (378.7) |
| Had first child | 126 | 256.8 (389.4) |  | 192.1 (322.5) |  | -64.7 (397.3) |  | 145 | 157.1 (288.1) |  | 69.9 (177.0) |  | -87.3 (285.8) |
| Same number of children | 80 | 371.5 (436.6) |  | 280.1 (373.7) |  | -91.3 (475.2) |  | 245 | 143.4 (291.3) |  | 187.6 (301.7) |  | 44.2 (389.3) |
| Had additional children | 81 | 274.3 (434.5) |  | 318.8 (401.4) |  | 44.5 (388.9) |  | 186 | 71.0 (189.4) |  | 57.7 (139.6) |  | -13.3 (193.8) |
| Transport PA (mins/wk) |  |  |  |  |  |  |  |  |  |  |  |  |  |
| Stayed child-free | 145 | 120.9 (168.3) |  | 132.3 (175.9) |  | 11.5 (223.9) |  | 213 | 123.5 (146.6) |  | 127.3 (169.0) |  | 3.8 (184.3) |
| Had first child | 126 | 125.5 (178.2) |  | 94.4 (135.5) |  | -31.1 (174.6) |  | 145 | 116.4 (171.9) |  | 110.9 (169.1) |  | -5.5 (213.7) |
| Same number of children | 80 | 111.0 (182.2) |  | 88.6 (108.1) |  | -22.4 (186.6) |  | 245 | 98.2 (156.2) |  | 78.1 (143.9) |  | -20.1 (198.5) |
| Had additional children | 81 | 62.5 (89.5) |  | 78.1 (127.4) |  | 15.5 (147.4) |  | 186 | 85.7 (125.7) |  | 103.4 (184.3) |  | 17.6 (202.6) |
| Domestic PA (mins/wk) |  |  |  |  |  |  |  |  |  |  |  |  |  |
| Stayed child-free | 145 | 137.8 (163.8) |  | 156.6 (194.3) |  | 18.8 (241.5) |  | 213 | 160.9 (183.3) |  | 162.7 (164.3) |  | 1.8 (190.7) |
| Had first child | 126 | 155.1 (157.7) |  | 195.5 (203.4) |  | 40.5 (222.9) |  | 145 | 165.7 (182.3) |  | 399.3 (368.1) |  | 233.6 (341.5) |
| Same number of children | 80 | 186.4 (175.2) |  | 199.8 (184.8) |  | 13.4 (218.1) |  | 245 | 455.9 (348.5) |  | 381.7 (298.0) |  | -74.2 (360.9) |
| Had additional children | 81 | 184.6 (178.8) |  | 222.0 (179.6) |  | 37.4 (166.7) |  | 186 | 368.0 (323.7) |  | 488.9 (351.1) |  | 120.9 (404.4) |
| Leisure time PA (LTPA, mins/wk) |  |  |  |  |  |  |  |  |  |  |  |  |  |
| Stayed child-free | 145 | 196.2 (247.0) |  | 215.1 (229.9) |  | 18.9 (252.3) |  | 213 | 209.1 (219.9) |  | 204.1 (197.5) |  | -5.0 (261.4) |
| Had first child | 126 | 212.7 (252.2) |  | 138.1 (176.6) |  | -74.7 (239.5) |  | 145 | 164.5 (168.9) |  | 124.6 (129.0) |  | -39.9 (179.0) |
| Same number of children | 80 | 161.5 (229.1) |  | 149.6 (172.7) |  | -11.9 (241.3) |  | 245 | 114.8 (147.4) |  | 154.5 (185.1) |  | 39.7 (183.3) |
| Had additional children | 81 | 132.6 (157.2) |  | 143.6 (162.0) |  | 11.0 (184.6) |  | 186 | 117.2 (145.4) |  | 143.9 (156.7) |  | 26.7 (175.6) |
| Sitting time (mins/day) |  |  |  |  |  |  |  |  |  |  |  |  |  |
| Stayed child-free | 145 | 387.6 (187.6) |  | 400.3 (186.9) |  | 12.7 (181.9) |  | 213 | 381.0 (153.0) |  | 397.9 (154.5) |  | 17.0 (157.7) |
| Had first child | 126 | 381.8 (172.3) |  | 380.5 (184.7) |  | -1.3 (194.2) |  | 145 | 390.4 (160.9) |  | 284.4 (148.7) |  | -106.0 (190.9) |
| Same number of children | 80 | 345.2 (171.6) |  | 315.7 (171.0) |  | -29.5 (173.4) |  | 245 | 268.0 (145.4) |  | 267.0 (142.4) |  | -1.0 (157.4) |
| Had additional children | 81 | 344.0 (183.4) |  | 323.8 (180.0) |  | -20.2 (148.5) |  | 186 | 289.3 (158.2) |  | 232.2 (139.3) |  | -57.1 (163.1) |
| TV viewing time (mins/day) |  |  |  |  |  |  |  |  |  |  |  |  |  |
| Stayed child-free | 145 | 132.8 (85.2) |  | 146.4 (109.7) |  | 13.6 (108.3) |  | 213 | 103.1 (69.6) |  | 110.2 (73.1) |  | 7.2 (81.7) |
| Had first child | 126 | 119.7 (95.2) |  | 115.4 (74.8) |  | -4.3 (95.4) |  | 145 | 100.4 (69.8) |  | 95.7 (70.7) |  | -4.7 (74.6) |
| Same number of children | 80 | 132.5 (84.8) |  | 126.2 (83.9) |  | -6.3 (91.9) |  | 245 | 107.2 (79.1) |  | 102.4 (73.0) |  | -4.8 (80.1) |
| Had additional children | 81 | 116.0 (98.3) |  | 117.6 (88.8) |  | 1.6 (91.1) |  | 186 | 95.9 (69.1) |  | 91.8 (60.2) |  | -4.0 (74.1) |

BMI, body mass index; CI, confidence interval; PA, physical activity; MVPA, moderate to vigorous intensity physical activity.

**Table S4** β (95% CI) for weight by change in parental status before or after removing women who had their baby in the last three months, Childhood Determinants of Adult Health Study, Australia, 1985-2011

|  | **Original results** | |  | **Removing women who had baby in the last 3 months** | |
| --- | --- | --- | --- | --- | --- |
|  | **n1** | **Adjusted model^a^** |  | **n2** | **Adjusted model^a^** |
|  |  | **β (95% CI)** |  |  | **β (95% CI)** |
| Weight (kg) |  |  |  |  |  |
| Stayed child-free | 309 | REF |  | 309 | REF |
| Had first child | 213 | **1.4 (0.1, 2.7)*** |  | 196 | 0.8 (-0.5, 2.1) |
| Same number of children | 382 | REF |  | 382 | REF |
| Had additional children | 212 | -0.8 (-1.9, 0.4) |  | 205 | -0.9 (-2.0, 0.3) |
| BMI (kg/m^2^) |  |  |  |  |  |
| Stayed child-free | 309 | REF |  | 309 | REF |
| Had first child | 213 | 0.5 (0, 1.0) |  | 196 | 0.3 (-0.2, 0.8) |
| Same number of children | 382 | REF |  | 382 | REF |
| Had additional children | 212 | -0.3 (-0.8, 0.1) |  | 205 | -0.4 (-0.8, 0.1) |

BMI, body mass index; CI, confidence interval.

^a^ Model was adjusted for age, sex, education and weight or BMI at baseline, follow-up length and marital transitions.

β (95% CI) in bold means statistically significant difference compared to the reference group (had first child born vs. stayed child-free, had additional children vs. same number of children).

**P*≤0.05.
